# Dose–Response Effects of Short-Term *Rhodiola rosea* (Golden Root Extract) Supplementation on Anaerobic Exercise Performance and Cognitive Function in Resistance-Trained Athletes: A Randomized, Crossover, Double-Blind, and Placebo-Controlled Study

**DOI:** 10.3390/nu17233736

**Published:** 2025-11-28

**Authors:** Majid S. Koozehchian, Andrew T. Newton, Gina Mabrey, Faith M. Bonness, Rafaela Rafajlovska, Alireza Naderi

**Affiliations:** 1 Department of Kinesiology, Jacksonville State University, Jacksonville, AL 36265, USA; atnewton@jsu.edu (A.T.N.); gmabrey@jsu.edu (G.M.); fbonness@stu.jsu.edu (F.M.B.); rrafajlovska@jsu.edu (R.R.); 2 Department of Sport Physiology, Boroujerd Branch, Azad University, Boroujerd 6915136111, Iran; naderi_a@yahoo.com

**Keywords:** *Rhodiola rosea*, ergogenic aids, adaptogens, sports nutrition, dietary supplements, exercise performance, resistance training, cognitive function

## Abstract

**Background:*** Rhodiola rosea* (RR) is marketed as an adaptogen; however, evidence for acute/short-term effects—and especially dose–response effects—in trained adults across performance and cognition is limited. **Objective:** Test whether RR improves resistance performance (strength, power) and executive function in resistance-trained adults using a randomized crossover with placebo and a capsule-free baseline. **Methods:** In a randomized, double-blind, crossover trial with four conditions—no-capsule control (CON), placebo (PL), low-dose RR (LDRR), and high-dose RR (HDRR). Twenty-seven resistance-trained adults completed the conditions. Day-7 testing included bench press (BP) and leg press (LP) 1-repetition maximum (1RM); a third set to failure at 60% 1RM with set-3 volume; Tendo mean/peak power; a 30 s Wingate; and the Stroop Color–Word Test. Secondary endpoints were Rating of Perceived Exertion (RPE), Readiness to perform using a Visual Analog Scale (VAS), and hemodynamics. **Results:** Versus CON, LDRR increased BP 1RM (+5.59 kg; *p* = 0.003), set-3 repetitions (+4.30; *p* < 0.001), set-3 volume (+168.6 kg; *p* < 0.001), and mean power (+29.7 W; *p* = 0.026). HDRR increased set-3 repetitions (+2.78; *p* = 0.005) and peak power (+34.2 W; *p* = 0.026), with a trend for set-3 volume (*p* = 0.086). LP 1RM exceeded CON with LDRR (+35.7 kg; *p* < 0.001) and HDRR (+47.7 kg; *p* < 0.001); contrasts vs. PL were significant. Wingate outcomes showed no consistent effects. Stroop improved vs. CON across all sections: Word +10.5 to +17.4 counts (*p* < 0.05), Color +6.1 to +12.0 (*p* ≤ 0.03), and Color–Word +10.2 to +18.9 (*p* < 0.001). **Conclusions:** Short-term RR consumption, regardless of dose or gender, improved resistance performance and significantly enhanced Stroop outcomes, with minimal changes in anaerobic cycling and RPE, and no consistent acute hemodynamic effects.

## 1. Introduction

*Rhodiola rosea* (RR) is a standardized herbal preparation containing rosavin, rosin, rosarin, and salidroside. The sports science literature characterizes its performance effects as modest and outcome-specific, rather than broadly ergogenic [1,2]. Because intake patterns can alter mechanisms and confound results, we distinguish between single pre-exercise dosing, short-term loading (3–7 days), and chronic continuous use (≥4 weeks) when summarizing prior work; the present trial targets short-term loading (7 days). In this study, “anaerobic” refers to short-duration, maximal cycling/sprint tests (e.g., 30 s Wingate), whereas “resistance” denotes external-load tasks (e.g., 1RM, bar velocity, repetitions to failure at %1RM); prior findings are organized by domain and test intensity, and we evaluate both.

Prior endurance trials are mentioned only to justify our inclusion of perceptual and hemodynamic measures: minor improvements on time-trial or time-to-exhaustion tasks have been reported alongside lower ratings of perceived exertion (RPE) and reduced heart rate (HR) [2,3,4,5]. For example, in double-blind crossover studies using 3 mg·kg^−1^, RR lowered submaximal HR and RPE during cycling and modestly improved a 6-mile time trial [3], without altering energy substrate use [4], consistent with a shift in effort perception rather than wholesale physiological change. In this study, we evaluated 7-day loading by administering the pre-test dose 60 min before testing on capsule days, thereby isolating short-latency effects rather than chronic effects.

Beyond steady-state endurance, RR has been tested in anaerobic and resistance contexts that directly align with our outcomes. With short loading strategies, physically active women produced higher mean/peak power, as well as total work, across repeated Wingate sprints (fatigue index unchanged), suggesting better power maintenance across bouts [6]. In resistance-trained adults, a comparable protocol increased mean bench press (BP) bar velocity (~8%) but reduced total repetitions to failure (~8%) within the same session, indicating a potential trade-off between movement speed and set-to-set endurance [7]. These patterns align with outcomes sensitive to power production and its maintenance across sets (Tendo-derived mean/peak power; set-3 reps/volume at 60% 1RM).

More recent randomized crossover work asked whether RR’s effects extend to upper-body strength when mental fatigue is induced. In a triple-blinded, four-session design (RR vs. placebo × control vs. mental-fatigue video), impact on visuo-cognitive performance and perceived exertion was trivial to small, with condition-dependent improvements in upper-body velocity or strength endurance—an outcome-specific rather than global pattern [8]. At the synthesis level, a systematic review (2022) similarly concluded that benefits, when they occur, are small and outcome-dependent [2].

Considered as a whole, the trial data point to modest effects that show up most clearly on predefined performance endpoints—for example, bar velocity or power during resistance sets and mean/peak power on the 30 s Wingate—even as small shifts in perceptual and hemodynamic measures often accompany these gains [6,7,8]. Methodological guidance emerging from reviews encourages the use of appropriate single-dose or short-loading strategies, careful reporting of extraction methods (including salidroside and rosavin content), and alignment of tasks with hypothesized mechanisms [1,2,9].

Mechanistically, RR bioactives—especially salidroside—may modulate monoaminergic (including putative MAO) pathways, β-endorphin pathways, and catecholaminergic pathways, thereby supporting fatigue resistance and power maintenance during repeated efforts [10,11,12]. Under denervation and nutrient-restriction conditions, salidroside consistently dampens oxidative stress and cytokine signaling, down-regulates MuRF1/MAFbx, and tempers proteostasis pathways involving the UPS and autophagy, thereby limiting loss of contractile proteins—features that would favor late-set power output [13,14]. At a systems level, the endurance of high-power work depends on AMPK–SIRT1–PGC-1α signaling and mitochondrial quality control. Preclinical studies indicate that salidroside activates these mechanisms, supporting mitochondrial biogenesis and stability [15,16,17]. The present study did not collect mechanistic biomarkers; these pathways are discussed only as a plausible context.

Findings on cognition are mixed. In models where RPE falls at matched workloads, executive performance on tasks such as the Stroop often changes little (trivial to small), even when upper-body velocity or strength-endurance shows condition-specific gains [8,18,19,20]. Measuring cognitive function alongside resistance and Wingate endpoints, therefore, helps localize any benefits—i.e., whether effects cluster around perception and power rather than global cognition [8].

Hemodynamic readouts are included primarily for mechanistic context and safety. Several human studies report a lower HR at matched submaximal workloads after RR [3,4], whereas consistent blood pressure changes are not evident in healthy, trained samples during brief tests [2]. Across clinical-style and sport trials summarized in recent reviews, no serious adverse events have been reported with typical dosing [2,9]. Consequently, we pre-specified HR, systolic blood pressure (SBP), and diastolic blood pressure (DBP) as secondary endpoints to aid interpretation of performance and perceptual outcomes.

Persistent methodological variability across prior work (e.g., extract identity, fixed vs. mg·kg^−1^ dosing, single-dose vs. loading, endurance vs. resistance vs. cognitive tasks) likely explains the divergent effects [1,2]. Designs that pre-specify performance endpoints and concurrently track perceptual (RPE/VAS) and hemodynamic (HR/SBP/DBP) measures are best positioned to clarify where RR’s small effects [1].

This randomized, within-subject four-period crossover (four conditions: capsule-free control, placebo, low-dose RR, high-dose RR; capsule arms double-blind) evaluated whether RR yields task-specific improvements in the co-primary endpoints of resistance performance (1RM; set-3 repetitions/volume at 60% 1RM; Tendo-derived mean/peak power) and executive function (Stroop), with secondary outcomes in anaerobic capacity (30 s Wingate: peak/mean power, total work, fatigue index), perceptual indices (RPE, VAS readiness), and hemodynamics (HR, SBP, DBP).

Our objective was to determine whether 7-day RR (200 or 1500 mg·day^−1^) improves resistance performance and executive function in resistance-trained adults compared to a placebo (and a capsule-free control for context). We hypothesized small to moderate, outcome-specific benefits within the double-blinded capsule arms (low/high dose > placebo), with larger effects for late-set volume/power than for the single-bout Wingate test, and minimal hemodynamic changes. Exploratory dose–response expectations were that the low dose favors strength-endurance and the high dose favors maximal strength/power. Recent syntheses reinforce this view. A 2023 systematic review of randomized trials [21] and a 2024 narrative review [1] conclude that RR’s ergogenic effects are generally minor and outcome-dependent, varying with dosing schedule, standardization, and task choice; a recent meta-analysis further reports improvements in endurance-related outcomes (e.g., VO_2_max, time-to-exhaustion) with heterogeneous effects across other domains [9].

## 2. Materials and Methods

### 2.1. Study Design, Oversight, and Reporting

**Figure 1:**
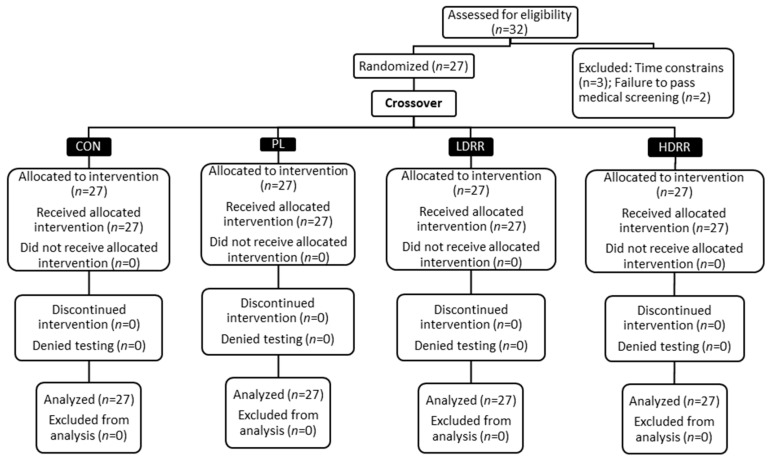
CONSORT participant flow: screening, randomization, allocation, follow-up, and analysis across CON, PL, LDRR, and HDRR (*n* = 27 analyzed per condition).

**Figure 2:**
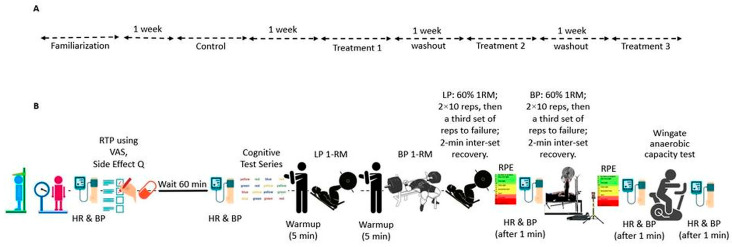
(**A**) Study timeline: familiarization; control (CON); and three randomized capsule periods—PL, LDRR, and HDRR—each separated by 7 days serving as the washout interval. (**B**) Visit flow (capsule periods): baseline HR, SBP, DBP→capsule dose→60 min seated (readiness-to-perform form + side-effect questionnaire)→SCWT→LP 1RM→BP 1RM→LP work sets→BP work sets→Wingate. HR, SBP, and DBP were assessed 1 min after set 3 of each lift and 1 min post-Wingate. For CON, no capsule was administered, and no 60 min post-dose interval occurred.

The trial was retrospectively registered at ClinicalTrials.gov (Identifier: NCT07225413) on 5 November 2025. A crossover, double-blind, randomized, placebo-controlled design was used in this investigation. All events took place in the Exercise Physiology and Nutrition Laboratory (EPNL) within the Department of Kinesiology at Jacksonville State University. The procedure was conducted in accordance with the Declaration of Helsinki and received approval from the institutional review board (IRB #01262022). The report follows the Consolidated Standards of Reporting Trials (CONSORT) guidelines for randomized crossover experiments [22]. Participants underwent four conditions: control (CON; no supplement), placebo (PL), low-dose RR (LDRR), and high-dose RR (HDRR). CON was scheduled as the first post-familiarization visit; the remaining capsule-based periods were completed in randomized order. Each period consisted of seven consecutive days under the assigned condition, with laboratory testing conducted on the seventh day. For the capsule periods, dosing was aligned with participants’ usual training times; the order of the capsule periods was randomized [3,6,7]. Figure 1 illustrates how participants were screened and assigned to a randomized sequence for PL/LDRR/HDRR, with a fixed period, with 1 CON kept in the study throughout. Figure 2 shows the four-period schedule and the test order for each visit. During CON, no capsule was administered, and testing proceeded without a pre-test seated interval; the 60 min pre-test interval applied only to capsule periods as depicted in Figure 2. We included a no-supplement CON to anchor a capsule-free, training-stable baseline and a capsule PL to isolate expectancy effects. CON was assigned to Period 1 to provide a capsule-free baseline; the three capsule periods (PL, LDRR, HDRR) were randomized using a balanced Williams design. Primary inferences were prespecified within the double-blinded capsule arms, thereby preserving randomization and counterbalancing for order/carryover effects. Because each participant completed all four periods, the crossover structure leverages within-subject comparisons to increase precision and power relative to a parallel design of similar size. To attenuate learning effects on the Stroop, participants completed a full practice administration during familiarization, using the same instructions and 45 s timing as on test days.

### 2.2. Participants and Eligibility

Participants were recruited through campus advertisements and email. Eligibility required individuals aged 18–40 years with at least 2 years of consistent resistance training experience [including BP and either leg press (LP) or squat]. Exclusion criteria comprised diagnosed metabolic, cardiovascular, or thyroid conditions; cardiac arrhythmias; active use of prescription drugs with possible cardiovascular or neurocognitive impacts; smoking; self-reported sensitivity to RR; regular alcohol consumption exceeding 12 drinks·week^−1^; and recent musculoskeletal injuries that might impede testing. Participants with resting heart rates (HR) >100 bpm, blood pressures ≥140/90 mmHg, DXA-derived body fat ≥30% (female) or ≥25% (male), current injury, pregnancy, or contraindications identified during familiarization were not included (see Section 2.3). A registered nurse obtained medical history and performed a brief physical examination during familiarization (see Section 2.3). To isolate the effects of RR, exclusion criteria also included current use of ergogenic dietary supplements or over-the-counter agents with plausible impacts on strength, power, cognition, or hemodynamics (e.g., creatine, beta-alanine, nitrate/beetroot products, stimulant capsules including caffeine pills, yohimbine, synephrine, and other adaptogens). Participants using such products were eligible only if they discontinued use and observed a ≥ 72 h washout period before each test day; habitual creatine users unwilling to suspend their intake were excluded. Routine micronutrients (e.g., multivitamins, vitamin D, or clinically indicated iron) were permitted if dose-stable across the study. Participants were resistance-trained (with a minimum of 2 years of experience); baseline 1RM BP and LP/squat were documented during familiarization.

### 2.3. Screening, Familiarization, and Standardization

Reference 1RM for BP and LP was established using a direct, incremental test; procedural details are provided in Section 2.7.1. Following telephone screening, eligible volunteers attended a familiarization visit to provide written informed consent, complete medical and training history questionnaires, and receive an overview of study procedures. During this visit, we measured stature, body mass, body composition, resting HR, SBP, and DBP. Height and body mass were measured using a digital physician scale with an integrated stadiometer (500KL, Health O Meter Professional; Pelstar LLC, McCook, IL, USA). Body mass was recorded to 0.1 kg, and stature to 0.01 m. Anthropometric evaluations adhered to recognized methods. Whole-body composition was measured with dual-energy X-ray absorptiometry (DXA; Hologic Horizon A, Hologic Inc., Marlborough, MA, USA). Scans were performed by the same technician, following the manufacturer’s guidance and the laboratory’s SOPs. After ≥10 min of quiet seated rest, resting HR, SBP, and DBP were recorded with an automated oscillometric monitor (Connex ProBP 3400, Welch Allyn, Skaneateles Falls, NY, USA). Subsequent visits were scheduled simultaneously to minimize circadian fluctuation, while ambient temperature and relative humidity were monitored during the visits.

Familiarization included one-repetition maximum (1RM) assessments for BP and LP [23] (standardized warm-ups; established strength-testing guidelines) and practice on the 30 s Wingate Anaerobic Test to attenuate learning effects before experimental testing. Participants were oriented to the Stroop Color–Word Test (SCWT) response format and instructed on completing the visual analog scale (VAS).

To standardize all testing sessions, participants were instructed to maintain a consistent sleep schedule, abstain from caffeine and alcohol, and avoid strenuous exercise for 48 h prior to testing. More stringent pre-visit restrictions for experimental test days are described in Section 2.6.

### 2.4. Randomization, Allocation Concealment, and Blinding

All participants completed CON as Period 1. The order of the three capsule-based conditions—PL, LDRR, and HDRR—across Periods 2–4 was randomized using a balanced 3 × 3 counterbalanced sequence to minimize period and order effects. Randomization sequences were generated by a computer program operated by a researcher not participating in data collection, and allocation was concealed using sequentially numbered, opaque, sealed envelopes prepared by an unbiased coordinator. Capsules for PL, LDRR, and HDRR were identical in appearance and mass and were provided in coded packaging; both participants and outcome evaluators were unaware of these capsule conditions. The CON visit could not be blinded at the participant level because no capsule was administered; to limit expectancy bias, standardized instructions and procedures were used across all visits.

### 2.5. Interventions and Dosing Regimen

Four periods were completed: CON (no supplement), PL (maltodextrin 1.5 g·day^−1^), LDRR (RR 200 mg·day^−1^ + maltodextrin 1.3 g·day^−1^), and HDRR (RR 1.5 g·day^−1^). Dose selection for LDRR and HDRR was informed by prior studies in athletes, which reported improvements in exercise performance and cognitive function with RR supplementation [6,7,24]. During PL, LDRR, and HDRR, participants received a single daily dose for seven consecutive days. On the laboratory test day, the final dose was administered on-site 60 min before testing, consistent with prior acute RR protocols [4,25]. Capsule periods were completed 7 days apart. RR extract powder (standardized to 3% salidroside and 1% rosavin) and maltodextrin were sourced from BulkSupplements.com (Henderson, NV, USA). Study products were stored in a temperature-controlled, low-light environment and dispensed in tamper-evident, coded containers. A 7-day washout was chosen a priori to exceed the expected elimination half-lives of RR bioactives and to allow any short-latency central/perceptual effects to resolve. This washout period is consistent with short-loading RR crossover work and, together with our counterbalanced sequence, was intended to minimize first-order carryover. Additionally, we enforced a 72 h supplement/OTC abstinence and a 48 h caffeine, alcohol, and exercise restriction period before each test day. The RR was a root extract from a single production lot (COA on file), standardized to 3% salidroside and containing rosavin congeners (rosavin, rosin, rosarin) as specified in the certificate of analysis. Over the 7-day periods, participants consumed 200 mg·day^−1^ (LDRR) or 1500 mg·day^−1^ (HDRR), yielding ≈6 mg·day^−1^ salidroside vs. ≈45 mg·day^−1^ salidroside, based on the stated standardization. Independent analytical verification (e.g., HPLC) was not conducted.

### 2.6. Pre-Visit Restrictions, Adherence, and Co-Interventions

Pre-visit instructions were standardized. In the 72 h preceding a laboratory visit, participants were instructed not to use dietary supplements or over-the-counter medications. For 48 h, they refrained from structured exercise, caffeine, alcohol, and mouth-rinsing products. Staff reviewed compliance at arrival and rescheduled noncompliant sessions. Capsule-period adherence (PL, LDRR, HDRR) was documented with daily dosing logs and returned-capsule counts. Participants were instructed not to initiate new training programs or other herbal/performance supplements across the study. At screening, we reviewed each participant’s supplement and OTC medication list and documented intended discontinuation plans. At every visit, staff verified adherence to the 72 h supplement/OTC and 48 h caffeine/alcohol/exercise restrictions; non-adherent sessions were rescheduled. Products prohibited during the 72 h window were those specified in Section 2.2.

### 2.7. Outcome Measures and Instruments

#### 2.7.1. Performance Testing

All performance testing was conducted under stable laboratory conditions to limit variability. Testing was conducted under the standardized pre-visit restriction and adherence protocol described in Section 2.6. On arrival, they completed a brief side-effect questionnaire and standard anthropometric checks. Strength testing targeted BP and LP. At every visit, a single 1RM verification was performed for both lifts to document day-to-day consistency; however, work-set loads were fixed at 60% of the 1RM established at familiarization and held constant across sessions. Participants then completed three work sets at that load with 2 min rests: sets 1 and 2 were fixed at 10 repetitions, and set 3 continued to volitional failure [26]. Performance volume was calculated as load × repetitions, summed across the three sets. During the BP, barbell displacement–time data were captured with a linear position transducer (Tendo Unit, Tendo Sport Machines, Trencín, Slovakia). From these data, the software computed mean power and peak power for each repetition; values were averaged across repetitions (set 3) to generate the set-level outcomes [27]. All strength assessments were conducted using standardized procedures on a flat bench and a hip/leg sled (Hammer Strength, Schiller Park, IL, USA) [28,29,30]. At each visit, a single 1RM verification was performed for BP and LP using the same direct protocol (warm-up ~5–10 reps at ~40–60% estimated 1RM, then 3–5 reps at ~60–80%; thereafter single attempts with 2.5–5 kg [BP] or 5–10 kg [LP] increments; 2–3 min rest for BP, 3–5 min for LP) until the maximal successful single with required range of motion was achieved (BP: bar to chest with controlled touch, full elbow extension; LP: controlled eccentric to ~90° knee flexion, full concentric return). Work-set loads were fixed at 60% of the familiarization 1RM and held constant across periods.

Anaerobic capacity was assessed with a 30 s Wingate on a calibrated friction-braked cycle ergometer (Monark 894E Peak Bike, Monark, Vansbro, Sweden). The relative braking force was set at 0.075 kg·kg^−1^ body mass. The warm-up consisted of five 5 s efforts at approximately 70 rpm, with easy pedaling between bouts. Afterward, participants performed a 30 s maximal sprint with standardized verbal encouragement to sustain effort [31,32].

#### 2.7.2. Cognitive and Subjective Assessments

Executive control was indexed with the SCWT in its complete paper-and-pencil form [33,34,35,36]. The assessment comprised three sequential sections: Word (read aloud color names printed in black), Color (state the font color of rows of “XXXX”), and Color–Word (state the font color of color words when the ink and the written word conflict, e.g., “RED” printed in blue). Each section was administered over a 45 s interval, with instructions prioritizing speed over accuracy. The score for each section was the count of correct responses completed within the 45 s window. Errors were noted, and the test manual was corrected on the spot. Our primary outcome was the total number of correct responses across all three sections; we also analyzed the scores for each section separately as a secondary measure. Immediately before testing, participants completed a VAS rating of muscle soreness, sleep quality, and related readiness items [37].

#### 2.7.3. Hemodynamic and Perceptual Responses; Tolerability

Resting HR and blood pressure were obtained after seated rest at check-in (see Section 2.3). Post-exercise HR and blood pressure were measured 1 min after BP set 3, 1 min after LP set 3, and 1 min after completion of the Wingate. All measurements were performed using the same device, posture, and cuff-selection procedures described in Section 2.3. At the 1 min post-exercise checkpoints, we also recorded Rating of Perceived Exertion (RPE; Borg 6–20). Tolerability was monitored only during supplemented sessions using a brief side-effect questionnaire; responses were documented in case-report forms. The questionnaire was not administered at familiarization or during CON because no supplement was provided [38].

### 2.8. Dietary Monitoring and Standardization

We asked participants to record their dietary intake in real time once per study week (4 total weeks) using MyFitnessPal. Each week, they completed a 3-day log (comprising two weekdays and one weekend day, consecutive when feasible). For each food and beverage, they documented the brand or manufacturer, preparation method, and quantity (in household measures or grams). Before Week 1, staff delivered brief, standardized training on portion estimation and app use.

### 2.9. Statistical Analysis

Data were analyzed using a four-period, within-subject crossover (CON, PL, LDRR, HDRR). For each endpoint, we fit a repeated-measures GLM with Treatment (4 levels) as the within-subject factor and Sex as a between-subjects factor, reporting the Treatment main effect and Treatment × Sex interaction. When the omnibus test was significant, we performed prespecified pairwise contrasts (PL, LDRR, HDRR vs. CON; LDRR vs. PL; HDRR vs. PL; HDRR vs. LDRR) with Bonferroni adjustment (two-tailed α = 0.05). As a sensitivity analysis, we also replicated the primary capsule-arm contrasts (PL, LDRR, HDRR) using linear mixed-effects models with subject-specific random intercepts and fixed effects for Treatment, Period, and Sex. These models yielded treatment effect estimates and *p*-values that were materially identical to those from the RM-GLM; therefore, only the RM-GLM estimates are presented in the tables and figures. Assumptions were checked using the Shapiro–Wilk test and residual diagnostics; Mauchly’s test was assessed with Greenhouse–Geisser corrections as needed. Descriptives are mean ± SD by sex; figures/tables present adjusted means ± SE (estimated marginal means) with 95% CIs, plotted relative to CON. Effect sizes for within-subject pairwise differences are Cohen’s dz (mean difference ÷ SD of paired differences) with thresholds: trivial <0.20, small 0.20–0.49, medium 0.50–0.79, large ≥0.80. Missing data were rare and handled by listwise deletion (no imputation). Analyses were performed in IBM SPSS Statistics v29.0 (IBM Corp., Armonk, NY, USA). PL vs. CON was evaluated as an expectancy/manipulation check. Power & carryover: with *n* = 27 completers, the crossover affords ≥80% power to detect dz ≈ 0.55–0.60 at α = 0.05 on prespecified capsule-arm contrasts; we therefore report Cohen’s dz with 95% CIs in lieu of post hoc power. Carryover, practice, and order effects were assessed via period/sequence terms and treatment-by-period diagnostics; no evidence of carryover/order was detected (all *p* > 0.10). Primary cognitive inferences used double-blinded contrasts (LDRR/HDRR vs. PL) to isolate effects beyond practice.

## 3. Results

### 3.1. Participants

Assumption checks were acceptable; period/sequence effects were not evident, and PL vs. CON showed no meaningful differences on key endpoints, supporting minimal expectancy and negligible carryover under the 7-day washout. Treatment × Sex interactions were non-significant for the co-primary endpoints; therefore, pooled estimates are presented, along with sex-stratified descriptives in Table 1. Analyses were conducted on participants who completed all four periods (*n* = 27). Enrollment and attrition are illustrated in Figure 1, while baseline characteristics are presented in Table 1. Additionally, diet logs revealed no between-period differences (Table 2). Sensitivity analyses using these linear mixed-effects models yielded treatment effects and *p*-values that were virtually identical to those from the RM-GLM, so RM-GLM estimates are reported throughout.

### 3.2. Primary Outcomes

#### 3.2.1. Exercise Performance

**Figure 3:**
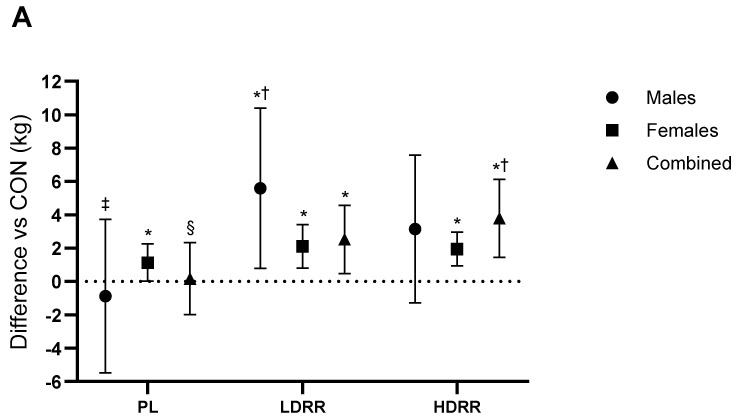

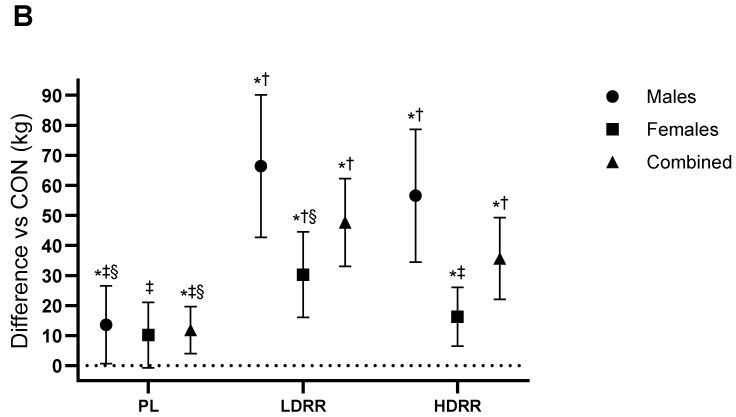
Changes in 1RM relative to the CON session. (**A**) Bench press; (**B**) Leg press. Points show adjusted means (±SE) with 95% CIs (error bars) for each treatment (CON, PL, LDRR, HDRR). Marker shapes are unique per condition. The dotted horizontal line represents no change vs. CON. Significance symbols: * vs. CON; † vs. PL; ‡ vs. LDRR; § vs. HDRR (Bonferroni-adjusted, two-tailed α = 0.05).

Bench press. 1RM (kg). A main ANOVA revealed that treatment influenced BP 1RM (Trt *p* = 0.001; Trt × G *p* = 0.08). Versus CON, PL was unchanged (−0.87 kg; 95% CI −5.48 to 3.73; *p* = 0.87; ES ≈ 0, trivial). LDRR exceeded CON by +5.59 kg (0.78–10.4; *p* = 0.003; +7.9% vs. CON; ES = 0.46, medium), whereas HDRR showed no clear difference (+3.15 kg; −1.29 to 7.58; *p* = 0.15; +4.4% vs. CON; ES = 0.28, small). LDRR > PL by 3.62 kg (1.19–6.05; *p* = 0.005; ES = 0.59, small–to–moderate), HDRR > PL by 2.36 kg (−0.06 to 4.77; *p* = 0.055; ES = 0.39, 0.24, small), and HDRR vs. LDRR −1.26 kg (−3.38 to 0.86; *p* = 0.23; ES = −0.24, trivial) (Figure 3A; Table 3).

**Figure 4:**
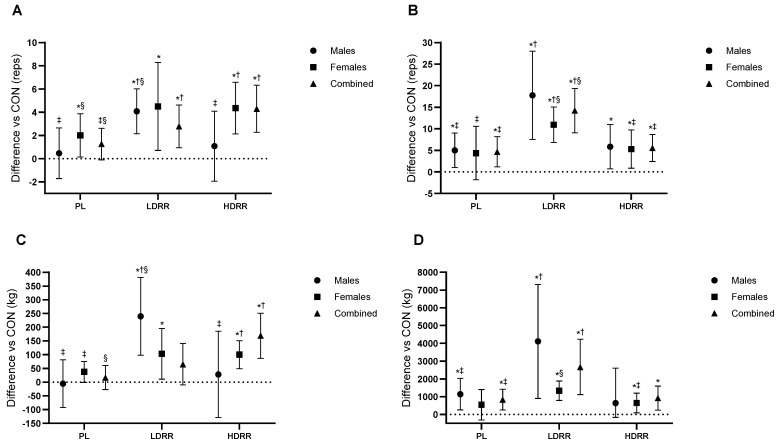
Set-3 performance relative to the CON session. (**A**) BP reps to failure at 60% 1RM; (**B**) LP reps to failure at 60% 1RM; (**C**) BP Set-3 LV; (**D**) LP Set-3 LV. Points show EMMs ± SE with 95% CIs (error bars) and slight horizontal jitter to avoid overlap; marker shapes are unique per condition with black outlines. Dotted line = no change vs. CON. Significance symbols: * vs. CON; † vs. PL; ‡ vs. LDRR; § vs. HDRR (Bonferroni-adjusted, two-tailed α = 0.05).

Set-3 repetitions to failure (60% 1RM). A primary ANOVA indicated a treatment effect (Trt *p* < 0.001; Trt × G *p* = 0.24). Versus CON, PL was not significantly different (+1.26 reps; 95% CI −0.10 to 2.62; *p* = 0.06; ES = 0.35, trivial–small). LDRR improved by +4.30 reps (2.27–6.33; *p* < 0.001; +39.6% vs. CON; ES = 0.84, large), and HDRR improved by +2.78 reps (0.93–4.62; *p* = 0.005; +25.6% vs. CON; ES = 0.60, moderate-to-large). Pairwise: LDRR > PL by 3.04 reps (1.09–4.98; *p* = 0.003; ES = 0.59, small–to–moderate), HDRR > PL by 1.52 reps (0.22–2.81; *p* = 0.023; ES = 0.44, small), and LDRR vs. HDRR +1.52 reps (−0.59 to 3.62; *p* = 0.15; ES = 0.27, trivial–small) (Figure 4A; Table 3).

Set-3 lifting volume (kg). ANOVA results confirmed treatment-related effects (Trt *p* < 0.001; Trt × G *p* = 0.039). Versus CON, PL was not different (+16.8 kg; 95% CI −26.8 to 60.4; *p* = 0.43; ES ≈ 0, trivial). LDRR increased volume by +168.6 kg (86.6–250.6; *p* < 0.001; +31.8% vs. CON; ES = 0.81, large), whereas HDRR showed no clear difference (+65.3 kg; −9.96 to 140.6; *p* = 0.086; +12.3% vs. CON; ES = 0.34, small–to–medium). LDRR > PL by 151.8 kg (70.5–233.1; *p* = 0.001; ES = 0.68, moderate), HDRR > PL by 48.5 kg (−1.14 to 98.2; *p* = 0.055; ES = 0.39, small), and LDRR vs. HDRR +103.2 kg (−0.37 to 206.9; *p* = 0.051; ES = 0.40, small–to–moderate) (Figure 4C; Table 3).

**Figure 5:**
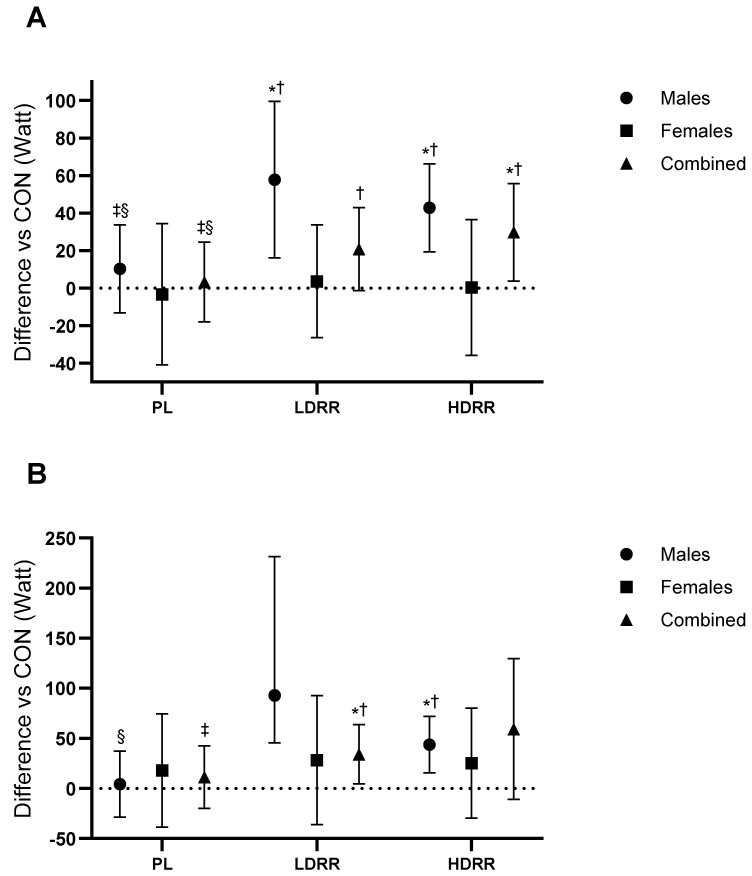
Tendo power relative to the CON session. (**A**) Mean power; (**B**) Peak power. Points show EMMs ± SE with 95% CIs (error bars); unique, outlined markers per condition; slight jitter applied. Dotted line = no change vs. CON. Significance symbols: * vs. CON; † vs. PL; ‡ vs. LDRR; § vs. HDRR (Bonferroni-adjusted, two-tailed α = 0.05). Units: watt.

Tendo Mean Power (W). The overall ANOVA revealed treatment effects and a significant interaction between Treatment and Gender (Trt *p* = 0.010; Trt × G *p* = 0.035). Versus CON, PL was not different (+3.33 W; 95% CI −17.8 to 24.5; *p* = 0.750; ES ≈ 0, trivial). LDRR exceeded CON by +29.7 W (3.81–55.7; *p* = 0.026; +14.6% vs. CON; ES = 0.45, medium), whereas HDRR was not significantly different (+20.8 W; −1.24 to 42.9; *p* = 0.06; +10.2% vs. CON; ES = 0.37, small–to–medium). LDRR > PL by 26.4 W (3.42–49.4; *p* = 0.026; ES = 0.45, small–to–moderate), HDRR > PL by 17.5 W (1.53–33.5; *p* = 0.033; ES = 0.42, small), and HDRR vs. LDRR 8.91 W (−5.76 to 23.5; *p* = 0.223; ES = 0.24, trivial–small) (Figure 5A; Table 3).

Tendo Peak Power (W). No treatment main effect (Trt *p* = 0.11; Trt × G *p* = 0.34). Versus CON, PL was not different (+11.4 W; 95% CI −19.8 to 42.6; *p* = 0.45; ES ≈ 0, trivial). LDRR was not significantly different (+59.3 W; −10.8 to 129.6; *p* = 0.09; +19.5% vs. CON; ES = 0.33, small–to–medium), and HDRR was higher by +34.2 W (4.51–63.9; *p* = 0.026; +11.2% vs. CON; ES = 0.46, medium). HDRR > PL by 22.7 W (4.69–40.8; *p* = 0.016; ES = 0.49, small–to–moderate), whereas LDRR vs. PL and HDRR vs. LDRR were not significant (+47.9 W; −17.2 to 113.1; *p* = 0.14; ES = 0.30, small–to–moderate) and (+25.2 W; −31.1 to 81.4; *p* = 0.36; ES = 0.18, small), respectively (Figure 5B; Table 3).

Leg press. 1RM (kg). ANOVA results confirmed a treatment-related effect on LP 1RM (Trt *p* < 0.001; Trt × G *p* < 0.001). Versus CON, PL was +11.8 kg (95% CI 4.02–19.7; *p* = 0.005; +3.8% vs. CON; ES = 0.10, trivial), LDRR was +35.7 kg (22.1–49.3; *p* < 0.001; +11.4% vs. CON; ES = 0.29, small), and HDRR was +47.7 kg (33.2–62.2; *p* < 0.001; +15.2% vs. CON; ES = 0.38, small–to–medium). LDRR > PL by 23.8 kg (10.2–37.5; *p* = 0.001; ES = 0.19, small), HDRR > PL by 35.8 kg (21.4–50.2; *p* < 0.001; ES = 0.28, small), and HDRR vs. LDRR +11.9 kg (−2.72 to 26.6; *p* = 0.11; ES = 0.09, trivial–small) (Figure 3B; Table 3).

Set-3 repetitions to failure (60% 1RM). A main treatment effect was present (Trt *p* < 0.001; Trt × G *p* = 0.34). Versus CON, PL was +4.67 reps (95% CI 1.17–8.16; *p* = 0.011; +19.2% vs. CON; ES = 0.42, small–to–moderate), HDRR was +5.56 reps (2.41–8.70; *p* = 0.001; +22.9% vs. CON; ES = 0.56, moderate), and LDRR produced the largest gain (+14.2 reps; 9.07–19.3; *p* < 0.001; +58.5% vs. CON; ES = 0.93, large). LDRR > PL by 9.56 reps (4.45–14.6; *p* = 0.001; ES = 0.61, moderate), LDRR > HDRR by 8.67 reps (2.57–14.7; *p* = 0.007; ES = 0.58, moderate), and PL vs. HDRR −0.89 reps (−4.11 to 2.33; *p* = 0.57; ES ≈ 0, trivial) (Figure 4B; Table 3).

Set-3 lifting volume (kg). Treatment effects were observed (Trt *p* = 0.006; Trt × G *p* = 0.14). Versus CON, PL was +832.6 kg (95% CI 245.9–1419.3; *p* = 0.007; +19.2% vs. CON; ES = 0.25, small), LDRR was +2670.8 kg (1116.7–4224.9; *p* = 0.002; +61.5% vs. CON; ES = 0.52, moderate), and HDRR was +921.2 kg (239.4–1603.0; *p* = 0.010; +21.2% vs. CON; ES = 0.31, small–to–moderate). LDRR > PL by +1838.2 kg (444.9–3231.4; *p* = 0.012; ES = 0.35, small–to–moderate), LDRR vs. HDRR +1749.6 kg (−130.0 to 3629.2; *p* = 0.06; ES = 0.35, small–to–moderate), and HDRR vs. PL +88.6 kg (−815.5 to 638.4; *p* = 0.80; ES = 0.03, trivial) (Figure 4D; Table 3).

Wingate anaerobic performance. No treatment effects were detected for any Wingate outcome—Peak power (Trt *p* = 0.54; Trt × G *p* = 0.45), Mean power (Trt *p* = 0.27; Trt × G *p* = 0.71), Total work (Trt *p* = 0.28; Trt × G *p* = 0.62), or Fatigue index (Trt *p* = 0.61; Trt × G *p* = 0.77). Pairwise contrasts among PL, LDRR, and HDRR versus CON were likewise non-significant (all *p* > 0.05). Adjusted means (±SE) showed only small, non-significant numerical differences across treatments—Peak power: 694, 706, 714, 691 W; Mean power: 505, 510, 523, 509 W; Total work: 14,621, 14,723, 15,168, 14,874 J; Fatigue index: 54.9, 55.8, 54.8, 53.1% (CON, PL, LDRR, HDRR). The main effect of Gender was observed for Peak power, Mean power, and Total work (all *p* < 0.001), but not for Fatigue index (*p* = 0.57). Complete estimates and contrasts are reported in Table 4.

#### 3.2.2. Effects on Cognitive Function

PL showed a slight improvement compared to CON, consistent with familiarization; however, both LDRR and HDRR exceeded PL on key Stroop outcomes (*p* < 0.05), indicating effects beyond practice. Consistent with the overall analyses, no Treatment × Sex interactions were detected for Stroop outcomes; results are therefore presented as a pooled analysis across sex.

**Figure 6:**
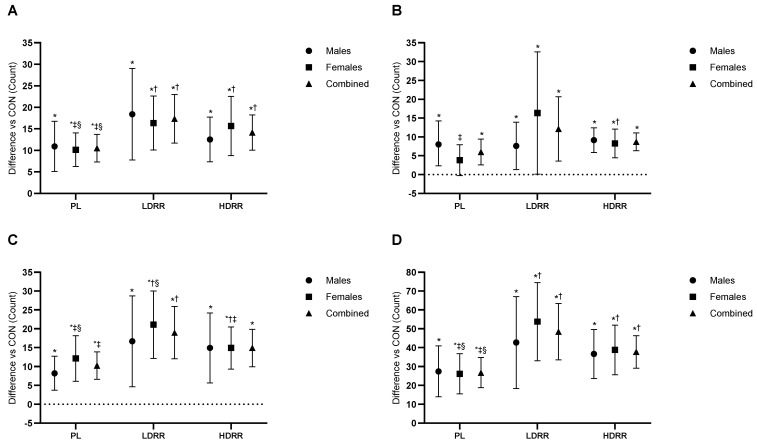
Stroop performance relative to the CON session. (**A**) Word; (**B**) Color; (**C**) Color–Word; (**D**) Total. Points show EMMs ± SE with 95% CIs (error bars); unique, outlined markers per condition; slight jitter applied. Dotted line = no change vs. CON. Significance symbols: * vs. CON; † vs. PL; ‡ vs. LDRR; § vs. HDRR (Bonferroni-adjusted, two-tailed α = 0.05). Units: counts.

Word. A primary ANOVA indicated a treatment effect on Word scores (Trt *p* < 0.001; Trt × G *p* = 0.63). Versus CON, PL was +10.5 items (95% CI 7.30–13.7; *p* < 0.001; +9.8% vs. CON; ES = 2.64, very large), LDRR was +14.1 items (10.1–18.3; *p* < 0.001; +13.1% vs. CON; ES = 2.80, very large), and HDRR was +17.3 items (11.7–22.9; *p* < 0.001; +16.1% vs. CON; ES = 2.49, very large). LDRR > PL by +3.63 items (0.45–6.81; *p* = 0.027; ES = 0.93, ~large) and HDRR > PL by +6.82 items (1.92–11.7; *p* = 0.008; ES = 1.13, large); HDRR vs. LDRR was +3.19 items (−1.94 to 8.31; *p* = 0.21; ES = 0.53, small–to–moderate) (Figure 6A; Table 5).

Color. A primary ANOVA indicated a treatment effect on Color scores (Trt *p* = 0.005; Trt × G *p* = 0.24). Versus CON, PL was +6.08 items (95% CI 2.70–9.46; *p* = 0.001; 7.7% vs. CON; ES = 0.71, medium), LDRR was +8.72 items (6.33–11.1; *p* < 0.001; 11.0% vs. CON; ES = 1.44, large), and HDRR was +11.9 items (3.45–20.5; *p* = 0.008; 15.1% vs. CON; ES = 0.56, medium). Among active treatments, LDRR > PL by +2.64 items (−0.03 to 5.31; *p* = 0.053; ES = 0.39, small), HDRR > PL by +5.90 items (−3.96 to 15.7; *p* = 0.23; ES = 0.24, small), and HDRR vs. LDRR was +3.27 items (−5.97 to 12.5; *p* = 0.47; ES = 0.14, trivial–small); none reached *p* < 0.05 (Figure 6B; Table 5).

Color–Word. A primary ANOVA indicated a treatment effect on Color–Word scores (Trt *p* < 0.001; Trt × G *p* = 0.72). Versus CON, PL was +10.1 items (95% CI 6.57–13.8; *p* < 0.001; 10.9% vs. CON; ES = 1.12, large), LDRR was +14.9 items (9.99–19.9; *p* < 0.001; 15.9% vs. CON; ES = 1.17, large), and HDRR was +18.8 items (11.8–25.9; *p* < 0.001; 20.1% vs. CON; ES = 1.06, large). LDRR > PL by +4.74 items (−0.28 to 9.76; *p* = 0.06; ES = 0.37, small), HDRR > PL by +8.70 items (2.85–14.5; *p* = 0.005; ES = 0.59, moderate), and HDRR vs. LDRR was +3.96 items (−2.26 to 10.1; *p* = 0.20; ES = 0.25, small); only HDRR > PL reached *p* < 0.05 (Figure 6C; Table 5).

Total. A primary ANOVA indicated a treatment effect on Total scores (Trt *p* < 0.001; Trt × G *p* = 0.723). Versus CON, PL was +26.7 items (95% CI 18.8–34.7; *p* < 0.001; 9.5% vs. CON; ES = 1.34, large), LDRR was +37.7 items (29.1–46.3; *p* < 0.001; 13.4% vs. CON; ES = 1.74, large), and HDRR was +48.4 items (33.4–63.4; *p* < 0.001; 17.2% vs. CON; ES = 1.28, large). Among active treatments, LDRR > PL by +11.0 items (3.39–18.6; *p* = 0.006; ES = 0.57, moderate), HDRR > PL by +21.6 items (6.31–37.0; *p* = 0.008; ES = 0.56, moderate), and HDRR vs. LDRR was +10.6 items (−4.12 to 25.4; *p* = 0.15; ES = 0.29, small); only LDRR > PL and HDRR > PL reached *p* < 0.05 (Figure 6D; Table 5).

### 3.3. Secondary Outcomes

#### 3.3.1. Perceptual Responses (VAS Readiness and RPE)

Visual Analog Scale—Readiness to Perform. VAS ratings of “Readiness to Perform” remained unchanged with treatment. Across the VAS models, the omnibus tests showed no treatment effect (Trt *p* = 0.12–0.97) and no Treatment × Gender interaction (Trt × G *p* = 0.23–0.85); adjusted means clustered tightly around ~3.5–4.0 units, with overlapping CIs, and none of the pairwise contrasts met *p* < 0.05.

RPE. A primary ANOVA showed no treatment effect for LP (Trt *p* = 0.24; Trt × G *p* = 0.06) or BP (Trt *p* = 0.96; Trt × G *p* = 0.69). RPE recorded after the third set clustered at a similarly high level (~15–16) across supplemented sessions (PL, LDRR, HDRR); pairwise contrasts were not significant (*p* ≥ 0.05).

#### 3.3.2. Hemodynamic Responses (Resting and 1 Min Post-Exercise HR and Blood Pressure)

Hemodynamic Summary. There were no significant treatment effects on resting or post-exercise hemodynamics. Resting HR and blood pressure did not differ by condition, and post-exercise HR and blood pressure were likewise non-significant across the BP, LP, and Wingate conditions. Overall, the interventions did not produce meaningful changes in HR or blood pressure.

#### 3.3.3. Analysis of Adverse Events

We monitored participants with comprehensive questionnaires that captured a wide spectrum of physical and psychological symptoms.

Side Effects Questionnaire: Across supplemented sessions, the side-effects index and all symptom-specific scores were statistically insignificant (all *p* > 0.05), and no Treatment × Gender effects emerged. Participants most often reported no or low-grade complaints; when symptoms did occur, they typically resolved within a short period. No serious adverse events were observed.

## 4. Discussion

To our knowledge, this is the first study to demonstrate that RR can elicit concurrent improvements in maximal strength, muscular endurance, and power—challenging a previously reported trade-off—while also producing dose-dependent nootropic effects at rest and decoupled perceptual responses in resistance-trained athletes. Specifically, this randomized, double-blind, within-subject crossover in resistance-trained adults tested two RR doses over 7 days across a multi-set resistance battery, a single-bout Wingate, and a cognitive task, using a salidroside-forward extract (3% salidroside; 1% rosavin (salidroside:rosavin, 3:1). No consistent sex differences were observed: Treatment × Sex interactions were non-significant for most endpoints, and where detected (e.g., LP 1RM, BP set-3 volume, Tendo mean power), effects were directionally similar in males and females and did not change the overall inferences. Because PL also improved compared to CON, we interpreted the Stroop findings primarily from the active-versus-placebo contrasts, which remained significant, supporting benefits beyond practice and order effects.

The resistance-exercise dose–response we observed—lower doses preferentially enhancing late-set muscular endurance/volume, and higher doses favoring maximal lower-body strength without sacrificing power—helps reconcile mixed findings by emphasizing test type (single-effort vs. repeated-effort), participant characteristics, dosing window, and extract chemistry. In trained men under a single-exercise, upper-body protocol, a 3-day load (1500 mg·day^−1^) + 500 mg pre-test (30–60 min) at 75% 1RM BP increased bar velocity but reduced repetitions to failure, indicating a same-session velocity–endurance trade-off in an isolated task [7]. By contrast, in physically active women performing repeated Wingates (3 × 15 s) with the same 3-day load and 500 mg pre-dose, mean/peak power, anaerobic capacity, and total work improved across bouts, indicating benefits emerged as fatigue accumulated [6]. Our protocol matches this fatigue-accumulating context, which likely explains the context-dependent synergy rather than a trade-off. To reduce confounding by chemistry, we used a salidroside-forward profile (~3:1) consistent with pharmacopeial/monograph specifications and widely studied preparations [6,7,39]; thus, remaining between-study differences are more plausibly driven by protocol design and dosing (e.g., short-term loading ≈3–7 days vs. longer courses). This interpretation aligns with recent syntheses concluding that RR’s effects are small and outcome-dependent, moderated by task selection, training status, dosing schedule, and standardization reporting [21]. As noted above, sex interactions were non-significant under our conditions.

No treatment effects were observed for Wingate anaerobic outcomes—peak power, mean power, total work, or fatigue index—indicating a task-specific response that favors multi-set resistance exercise over single-bout anaerobic power. A previous study reported that short-term RR supplementation significantly improved mean power, peak power, and total work in physically active women who performed three 15 s Wingate bouts after three days of loading [6]. Taken together, these results suggest that RR may enhance anaerobic performance across repeated bouts of anaerobic exercise, but not single bouts. It remains plausible that RR enhances power maintenance and fatigue resistance across repeated high-intensity efforts, but does not enhance absolute peak power or capacity in a fresh, non-fatigued state. This contrast is compatible with protocols that emphasize repeated supramaximal efforts—where power maintenance across bouts is taxed [6]—rather than a single 30 s sprint; in other words, conditions that mirror our late-set resistance outcomes are more sensitive than isolated, fresh sprints [40,41].

A short-latency CNS mechanism most plausibly explains the present effects (7-day exposure; gains in late-set resistance and Stroop). Evidence that RR modulates monoaminergic pathways (including putative MAO effects), β-endorphins, and catecholamines—on timeframes of hours to days—supports this interpretation and maps onto outcomes governed by effort regulation, central motor drive, and power maintenance across sets [39,42]. By contrast, mitochondrial biogenesis and other structural adaptations typically require multi-week training; they are a plausible long-term hypothesis, but were not tested by our 7-day design [43,44].

Integrating the resistance, Wingate, and cognitive results also aligns with a short-latency CNS/neuromuscular explanation: cognitive benefits and late-set resistance gains co-occurred, whereas a single-bout Wingate—less dependent on central regulation across repeated efforts—showed no consistent change, mirroring prior RR findings in repeated Wingates vs. single-exercise bench-press contexts [6,7].

In our study, cognitive performance improved significantly after 7 days of RR in a clear dose-responsive pattern. These effects align with prior reports of improved attention, processing speed, and fatigue resistance under stress/fatigue [18,19,20,22,23] and, notably, emerged here in a rested, resistance-trained cohort with large effect sizes, underscoring the robust nootropic potential. A central mechanism is plausible, including monoamine oxidase inhibition [10] and/or modulation of neuropeptides [45,46].

This study observed no significant treatment effects on RPE during BP or LP. Likewise, there were no significant effects on resting or 1 Min post-exercise HR, SBP, and DBP across exercise modalities, or on readiness to perform. This contrasts with endurance-focused studies, where RR reduces RPE and HR [4,23,46], indicating modality specificity and placing our findings in the minority. However, these inconsistencies can be explained through our timescale model: acute CNS effects (e.g., MAO inhibition [10], cortisol modulation [45,47]) may enhance resistance performance without altering perception or hemodynamics, unlike endurance protocols where oxidative/anti-fatigue mechanisms dominate. The absence of changes fits rapid-onset pathways rather than chronic ones, consistent with the overall results. The discrepancy with endurance-focused findings likely reflects task modality and internal-load duration (brief, high-intensity sets versus sustained workloads).

Improvements with LDRR—e.g., bench-press set-3 repetitions +39.6% and volume +31.8%, leg-press repetitions +58.5%, and Tendo mean power +14.6%—are actionable magnitudes for trained athletes, as they influence weekly volume-load progression and late-set power retention. Stroop gains of ~9–20% indicate meaningful enhancement of executive control under standardized conditions. Given the within-subject design, fixed %1RM work-set loads, and on-site standardization, these effects are unlikely to reflect day-to-day noise and are consistent with performance-relevant changes.

Practically, these findings suggest that 7-day RR supplementation (200–1500 mg/day) may benefit athletes in resistance training programs that emphasize volume and fatigue resistance, such as those used in hypertrophy or strength protocols [23,26,28]. The lack of Wingate effects implies limited utility for single-bout explosive sports (e.g., sprinting), though repeated-effort contexts (e.g., team sports) may benefit [6,8]. The cognitive enhancements further support RR for sports requiring mental acuity alongside physical demands. Overall, benefits appear to depend on rapid-onset CNS/neuromuscular mechanisms. The absence of side effects supports RR’s tolerability, consistent with prior trials [1,2].

Limitations include the sample size, which may have limited power to detect subtle Wingate effects, and the focus on trained individuals (2+ years of resistance experience), potentially limiting generalizability to untrained or female-only cohorts, where sex-specific responses have been noted [6]. The 7-day duration bridges acute and short-term protocols but does not address chronic effects; longer trials are needed to confirm the absence of chronic adaptations. While Tendo power measurements were reliable [27], the addition of biomarkers (e.g., oxidative stress, inflammatory markers [24,32]) could help clarify the mechanisms. Additionally, the CON period was always first and not participant-blinded, which may have introduced expectancy/order effects; we mitigated this by counterbalancing the capsule periods with standardizing instructions. The crossover design [22] minimized bias, but testing in fatigued states or with adjuncts (e.g., caffeine [31]) could enhance ecological validity. While CON was not participant-blinded, standardized procedures and the observed PL ≈ CON on key variables argue against material expectancy effects; primary inferences relied on double-blinded capsule-arm comparisons. Composition relied on the manufacturer’s certificate of analysis; no third-party assay (e.g., HPLC) or contaminant screening was performed, which limits external validity. We refer to biological sex (male/female) when considering effect modification; pooled inferences were retained given no Treatment × Sex interaction.

Sex distribution was balanced (14 women, 13 men); no Treatment × Sex interactions were detected for co-primary endpoints, so pooled inferences were retained. Given the sample size, sex-by-dose effects remain underpowered and warrant prospective power analyses; interaction *p*-values are reported alongside the models.

For resistance-trained athletes, a short loading phase (7 days) with the final dose ~60 min pre-session is a pragmatic strategy. A dose of 200 mg·day^−1^ may be prioritized when the goal is late-set volume/strength-endurance and mean power, whereas a dose of 1500 mg·day^−1^ may better support maximal lower-body strength and peak power; both doses improved executive performance in our cohort. Effects were task-specific (no consistent benefit for a single-bout Wingate). We observed no significant changes in HR or BP, and no serious adverse events occurred in healthy, trained adults. Contraindications/precautions: avoid concomitant monoamine oxidase inhibitors; use caution with serotonergic medications, arrhythmias, pregnancy/lactation, and unstable endocrine conditions. The selection of lot-documented products and, when feasible, third-party testings are recommended.

## 5. Conclusions

This randomized, crossover trial shows that 7-day supplementation with *Rhodiola rosea* (200–1500 mg/day) causes dose-dependent improvements in resistance exercise performance—enhancing strength, endurance, and power without trade-offs—and produces significant nootropic effects on cognitive function in rested, resistance-trained athletes, while separating these benefits from perceptual or hemodynamic changes. These results, not observed in single-bout anaerobic tasks, highlight rapid CNS and neuromuscular mechanisms over long-term mitochondrial adaptations, aligning with emerging evidence on RR’s ergogenic potential. Practically, RR may optimize training strategies focused on fatigue resistance and mental clarity, with excellent tolerability. Future research should investigate longer durations, biomarkers, and different populations to improve the application.

## Figures and Tables

**Table 1:** Baseline demographics.

Measurement	Total	Male	Female	*p*-Value
*n*	27	13	14	
Age (years)	22.3 ± 3.78	23.0 ± 5.33	21.7 ± 1.25	0.09
Height (cm)	170.3 ± 10.7	178.2 ± 9.47	163.1 ± 5.32	0.08
Weight (kg)	75.4 ± 21.9	91.3 ± 15.5	60.6 ± 15.9	0.92
Body Mass Index (kg/m^2^)	25.7 ± 6.24	28.8 ± 4.96	22.8 ± 6.02	0.77
Bench press 1RM (kg)	67.7 ± 39.8	101.1 ± 29.9	36.7 ± 13.6	0.51
Leg press 1RM (kg)	290.3 ± 117.6	391.2 ± 83.5	196.5 ± 39.9 ^†^	0.01

Data are mean ± SD. *p*-values reflect male vs. female comparisons. ^†^ denotes significantly different from males (*p* < 0.05).

**Table 2:** Dietary analysis.

Variable	Treatment	Total	Male	Female	*p*-Value
Energy Intake	CON	1702.3 ± 580.5	1962.5 ± 687.4	1460.7 ± 327.4	0.58
	PL	1795.5 ± 594.9	2234.1 ± 512.7	1388.4 ± 307.2	
	LDRR	1660.8 ± 545.8	2024.2 ± 517.3	1323.4 ± 307.0	
	HDRR	1721.7 ± 569.7	2029.9 ± 594.3	1435.5 ± 374.3	
Protein	CON	88.4 ± 44.3	112.1 ± 47.2	66.5 ± 28.2	0.96
	PL	90.5 ± 40.8	114.1 ± 40.6	68.6 ± 27.2	
	LDRR	88.4 ± 40.8	112.3 ± 42.2	66.2 ± 24.1	
	HDRR	88.2 ± 41.8	110.3 ± 50.1	67.6 ± 15.6	
Carbohydrate	CON	187.1 ± 44.3	207.0 ± 46.1	168.6 ± 34.8	0.22
	PL	182.0 ± 74.3	214.1 ± 83.8	152.1 ± 50.6	
	LDRR	169.7 ± 49.6	190.4 ± 52.8	150.6 ± 39.0	
	HDRR	172.8 ± 57.9	191.4 ± 59.3	155.6 ± 53.0	
Fat	CON	79.7 ± 53.8	106.0 ± 64.5	55.2 ± 24.9	0.09
	PL	73.8 ± 31.5	92.8 ± 33.8	56.3 ± 15.6	
	LDRR	63.9 ± 31.9	82.5 ± 34.3	46.6 ± 16.8	
	HDRR	69.5 ± 26.3	82.5 ± 26.6	57.3 ± 20.2	

Values are the mean ± SD from 3-day food logs for each condition. Energy intake is reported as kcal/day; macronutrients as g/day.

**Table 3:** Resistance Exercise Performance.

Variable	Treatment	Male Mean ± SD	Female Mean ± SD	Mean ± SE	*p*-Value	
BP 1RM (kg)	CON	103.1 ± 29.5^c^	38.6 ± 13.8 ^b,c,d^	70.8 ± 4.40 ^c,d^	Trt	0.001 ***
	PL	102.2 ± 30.6^c^	39.7 ± 13.3 ^a^	71.0 ± 4.50 ^c,d^	Trt × G	0.08
	LDRR	108.7 ± 30.4 ^a,b^	40.7 ± 13.7 ^a^	74.7 ± 4.50 ^a,b^		
	HDRR	106.2 ± 32.1	40.5 ± 13.4 ^a^	73.4 ± 4.67 ^a,b^		
LP 1RM (kg)	CON	409.1 ± 85.1 ^b,c,d^	226.5 ± 42.6 ^c,d^	314.4 ± 113.5 ^a,b,c^	Trt	<0.001 ***
	PL	422.7 ± 89.3 ^a,c,d^	236.7 ± 45.6 ^c^	326.2 ± 117.0 ^a,c,d^	Trt × G	0.001 ***
	LDRR	475.4 ± 101.4 ^a,b^	256.8 ± 44.3 ^a,b,d^	362.1 ± 134.6 ^a,b^		
	HDRR	465.6 ± 101.4 ^a,b^	242.8 ± 42.5 ^a,c^	350.1 ± 136.1 ^a,b^		
BP Reps to Failure S3 @ 60% 1RM (reps)	CON	12.3 ± 5.37^c^	9.36 ± 3.62 ^b,c,d^	10.8 ± 0.87 ^c,d^	Trt	<0.001 ***
	PL	12.8 ± 5.22^c^	11.3 ± 3.91 ^a,d^	12.1 ± 0.88 ^c,d^	Trt × G	0.24
	LDRR	16.4 ± 6.65 ^a,b,d^	13.8 ± 6.52 ^a^	15.1 ± 1.26 ^a,b^		
	HDRR	13.4 ± 5.41^c^	13.7 ± 4.42 ^a,b^	13.5 ± 0.94 ^a,b^		
LP Reps to Failure S3 @ 60% 1RM (reps)	CON	22.9 ± 12.9 ^b,c,d^	25.7 ± 8.03 ^b,c,d^	24.3 ± 2.06 ^b,c,d^	Trt	<0.001 ***
	PL	27.9 ± 12.2 ^a,c^	30.8 ± 11.4 ^a,d^	28.9 ± 2.28 ^a,c^	Trt × G	0.34
	LDRR	40.6 ± 25.8 ^a,b^	36.6 ± 9.50 ^a^	38.6 ± 3.69 ^a,b,d^		
	HDRR	28.7 ± 10.6 ^a^	31.0 ± 7.81 ^a,b^	29.8 ± 1.78 ^a,c^		
BP—S3 Lifting Volume (kg)	CON	819.4 ± 437.6 ^c^	239.6 ± 145.3 ^c,d^	518.8 ± 431.4 ^c^	Trt	<0.001 ***
	PL	813.8 ± 499.5 ^c^	277.2 ± 118.9 ^d^	535.6 ± 443.7 ^c^	Trt × G	0.039 *
	LDRR	1058.6 ± 642.6 ^a,b,d^	342.7 ± 201.1 ^a^	687.4 ± 586.2 ^a,b,d^		
	HDRR	847.5 ± 528.9 ^c^	339.6 ± 152.9 ^a,b^	584.1 ± 455.7 ^c^		
LP—S3 Lifting Volume (kg)	CON	5606.1 ± 4050.0 ^b,c^	3081.5 ± 1295.1 ^c,d^	4297.1 ± 3171.9 ^b,c,d^	Trt	0.006 **
	PL	6747.5 ± 4068.3 ^a,c^	3627.4 ± 1834.2 ^a,c^	5129.7 ± 3441.6 ^a,c^	Trt × G	0.14
	LDRR	9713.4 ± 8549.2 ^a,b^	4418.4 ± 1749.9 ^a,d^	6967.9 ± 6521.7 ^a,b^		
	HDRR	6826.1 ± 2942.9	3725.3 ± 1372.6 ^a,c^	5218.3 ± 2726.1^a^		
Tendo Mean Power (Watt)	CON	291.9 ± 104.4 ^c,d^	116.0 ± 69.5	204.0 ± 16.9 ^c,d^	Trt	0.010 *
	PL	302.3 ± 106.3^c^	112.7 ± 43.3	207.5 ± 15.4 ^c,d^	Trt × G	0.035 *
	LDRR	349.8 ± 120.2 ^a,b^	119.7 ± 42.5	234.7 ± 17.1 ^a,b^		
	HDRR	334.8 ± 116.4^a^	116.4 ± 41.4	225.6 ± 16.5 ^a,b^		
Tendo Peak Power (Watt)	CON	459.3 ± 196.4	151.1 ± 83.8	305.1 ± 28.6	Trt	0.11
	PL	463.6 ± 175.2	169.1 ± 75.1	316.3 ± 25.6	Trt × G	0.34
	LDRR	552.1 ± 321.5	179.3 ± 98.7	365.7 ± 45.0		
	HDRR	503.1 ± 203.7	176.3 ± 76.7	339.7 ± 29.2		

Data are means ± SD. RM-GLM results are reported as *p*-values for Treatment (Trt) and Treatment × Gender (Trt × G). * *p* < 0.05, ** *p* < 0.01, *** *p* < 0.001. ^a^ denotes a significant difference from CON. ^b^ denotes a significant difference from PL. ^c^ denotes a significant difference from LDRR. ^d^ denotes a significant difference from HDRR.

**Table 4:** Wingate anaerobic performance.

Variable	Treatment	Male Mean ± SD	Female Mean ± SD	Mean ± SE	*p*-Value	
Peak Power (W)	CON	851.4 ± 138.7	536.9 ± 120.8	694.1 ± 24.9	Trt	0.54
	PL	854.2 ± 149.1	557.4 ± 108.1	705.8 ± 24.9	Trt × G	0.45
	LDRR	838.1 ± 140.9	544.3 ± 121.6	691.2 ± 25.2		
	HDRR	885.9 ± 183.4	541.6 ± 111.4	713.7 ± 28.9		
Mean Power (W)	CON	627.9 ± 102.5	382.9 ± 79.7	505.4 ± 17.5	Trt	0.277
	PL	634.7 ± 123.5	385.2 ± 77.8	510.0 ± 19.7	Trt × G	0.71
	LDRR	634.2 ± 131.5	383.2 ± 86.1	508.7 ± 21.2		
	HDRR	656.3 ± 114.6	390.1 ± 80.5	523.2 ± 18.9		
Total Work (J)	CON	18202.1 ± 3246.8	11,039.1 ± 2341.6	14,620.6 ± 541.6	Trt	0.287
	PL	18,270.6 ± 4083.2	11,176.2 ± 2306.1	14,723.4 ± 631.9	Trt × G	0.62
	LDRR	18,448.6 ± 4197.6	11,299.2 ± 2344.5	14,873.9 ± 647.8		
	HDRR	19,057.7 ± 3452.1	11,278.6 ± 2413.5	15,168.2 ± 569.6		
Fatigue Index (%)	CON	53.1 ± 15.7	56.7 ± 12.2	54.9 ± 2.71	Trt	0.609
	PL	54.0 ± 15.3	57.5 ± 12.3	55.7 ± 2.67	Trt × G	0.77
	LDRR	50.9 ± 18.4	55.2 ± 12.4	53.1 ± 3.01		
	HDRR	54.6 ± 19.2	54.9 ± 13.2	54.7 ± 3.16		

Data are means ± SD. RM-GLM results are reported as *p*-values for Treatment (Trt) and Treatment × Gender (Trt × G).

**Table 5:** Cognitive Function.

Variable	Treatment	Male ± SD	Female ± SD	Mean ± SE	*p*-Value	
Word (counts)	CON	104.5 ± 11.9	110.9 ± 8.49	107.7 ± 1.98 ^b,c,d^	Trt	<0.001 ***
	PL	115.4 ± 15.5	121.1 ± 5.98	118.2 ± 2.23 ^a,c,d^	Trt × G	0.63
	LDRR	117.1 ± 17.2	126.5 ± 11.5	121.8 ± 2.81 ^a,b^		
	HDRR	122.9 ± 22.2	127.2 ± 7.84	125.1 ± 3.16 ^a,b^		
Color (counts)	CON	75.6 ± 12.2	82.6 ± 11.8	79.1 ± 2.31 ^b,c,d^	Trt	0.03 *
	PL	84.0 ± 10.4	86.5 ± 11.1	85.2 ± 2.07 ^a^	Trt × G	0.24
	LDRR	84.8 ± 10.7	90.9 ± 11.0	87.8 ± 2.1 ^a^		
	HDRR	83.3 ± 18.1	99.0 ± 28.2	91.1 ± 4.61 ^a^		
Color- Word (counts)	CON	92.3 ± 17.2	95.6 ± 20.1	93.9 ± 3.62 ^b,c,d^	Trt	<0.001 ***
	PL	100.5 ± 17.5	107.7 ± 14.7	104.1 ± 3.11 ^a,d^	Trt × G	0.73
	LDRR	107.2 ± 25.3	110.5 ± 15.9	108.9 ± 4.04 ^a^		
	HDRR	109.0 ± 23.8	116.7 ± 10.6	112.8 ± 3.51 ^a,b^		
Total Stroop Results (counts)	CON	272.5 ± 34.8	289.2 ± 25.8	280.8 ± 5.87 ^b,c,d^	Trt	<0.001 ***
	PL	300.0 ± 37.6	315.3 ± 18.2	307.6 ± 5.63 ^a,c,d^	Trt × G	0.63
	LDRR	309.1 ± 46.4	328.1 ± 22.4	318.6 ± 6.93 ^a,b^		
	HDRR	315.2 ± 58.7	343.0 ± 31.5	329.1 ± 8.98 ^a,b^		

Data are means ± SD. RM-GLM results are reported as *p*-values for Treatment (Trt) and Treatment × Gender (Trt × G). * *p* < 0.05, *** *p* < 0.001. ^a^ denotes a significant difference from CON. ^b^ denotes a significant difference from PL. ^c^ denotes a significant difference from LDRR. ^d^ denotes a significant difference from HDRR.

## Data Availability

Data are contained within the article.

## Abbreviations

The following abbreviations are used in this manuscript:

RR	*Rhodiola rosea*
CON	Control
PL	Placebo
LDRR	Low-dose *Rhodiola rosea*
HDRR	High-dose *Rhodiola rosea*
HR	Heart rate
SBP	Systolic blood pressure
DBP	Diastolic blood pressure
RPE	Rating of perceived exertion
BP	Bench press
LP	Leg press
VAS	Visual analog scale
SCWT	Stroop color word test
1RM	1 Repetition maximum
TRT	Treatment
G	Gender
